# Deep learning segmentation model for quantification of infarct size in pigs with myocardial ischemia/reperfusion

**DOI:** 10.1007/s00395-024-01081-x

**Published:** 2024-09-30

**Authors:** Felix Braczko, Andreas Skyschally, Helmut Lieder, Jakob Nikolas Kather, Petra Kleinbongard, Gerd Heusch

**Affiliations:** 1 Institute for Pathophysiology, West German Heart and Vascular Center, University of Duisburg-Essen, Hufelandstr. 55, 45122 Essen, Germany; 2 Department of Medicine III, University Hospital RWTH Aachen, Aachen, Germany; 3 Else Kroener Fresenius Center for Digital Health, Medical Faculty Carl Gustav Carus, Technical University Dresden, Dresden, Germany

**Keywords:** Deep learning, Infarct size, Myocardial ischemia/reperfusion, Pig, Segmentation

## Abstract

Infarct size (IS) is the most robust end point for evaluating the success of preclinical studies on cardioprotection. The gold standard for IS quantification in ischemia/reperfusion (I/R) experiments is triphenyl tetrazolium chloride (TTC) staining, typically done manually. This study aimed to determine if automation through deep learning segmentation is a time-saving and valid alternative to standard IS quantification. High-resolution images from TTC-stained, macroscopic heart slices were retrospectively collected from pig experiments (*n* = 390) with I/R without/with cardioprotection to cover a wide IS range. Existing IS data from pig experiments, quantified using a standard method of manual and subsequent digital labeling of film-scan annotations, were used as reference. To automate the evaluation process with the aim to be more objective and save time, a deep learning pipeline was implemented; the collected images (*n* = 3869) were pre-processed by cropping and labeled (image annotations). To ensure their usability as training data for a deep learning segmentation model, IS was quantified from image annotations and compared to IS quantified using the existing film-scan annotations. A supervised deep learning segmentation model based on dynamic U-Net architecture was developed and trained. The evaluation of the trained model was performed by fivefold cross-validation (*n* = 220 experiments) and testing on an independent test set (*n* = 170 experiments). Performance metrics (Dice similarity coefficient [DSC], pixel accuracy [ACC], average precision [mAP]) were calculated. IS was then quantified from predictions and compared to IS quantified from image annotations (linear regression, Pearson’s *r*; analysis of covariance; Bland–Altman plots). Performance metrics near 1 indicated a strong model performance on cross-validated data (DSC: 0.90, ACC: 0.98, mAP: 0.90) and on the test set data (DSC: 0.89, ACC: 0.98, mAP: 0.93). IS quantified from predictions correlated well with IS quantified from image annotations in all data sets (cross-validation: *r* = 0.98; test data set: *r* = 0.95) and analysis of covariance identified no significant differences. The model reduced the IS quantification time per experiment from approximately 90 min to 20 s. The model was further tested on a preliminary test set from experiments in isolated, saline-perfused rat hearts with regional I/R without/with cardioprotection (*n* = 27). There was also no significant difference in IS between image annotations and predictions, but the performance on the test set data from rat hearts was lower (DSC: 0.66, ACC: 0.91, mAP: 0.65). IS quantification using a deep learning segmentation model is a valid and time-efficient alternative to manual and subsequent digital labeling.

**Supplementary Information:**

The online version contains supplementary material available at 10.1007/s00395-024-01081-x.

## Introduction

Preclinical studies on mechanical and pharmacological cardioprotective interventions have been largely positive and promising, but their translation to clinical practice to achieve a benefit for patients with myocardial infarction has been mostly disappointing [13, 15, 18, 29]. This translational gap has been attributed to clinical studies in patients who regularly have advanced age, co-morbidities and co-medications and to preclinical studies in animals, which are usually young and healthy [14, 23]. In preclinical studies, there is often a lack of robustness and there are only few neutral studies [53]. Preclinical data may differ between centers [49], and reproducibility and rigor are of concern in cardioprotection research and in the biomedical community in general [7, 12], despite the existence of established guidelines [3, 22, 29, 37]. Infarct size (IS) is the most robust end point for assessing cardioprotective efficacy [3], since IS is the decisive determinant for survival in patients with acute myocardial infarction [59]. The recommended method for quantification of IS in patients is cardiac magnetic resonance imaging (cMRI), while for preclinical studies it is histochemistry by triphenyl tetrazolium chloride (TTC) staining in combination with the delineation of the area at risk (AAR) with use of a blue or fluorescent dye [3, 16, 21]. Here, the delineation and demarcation of the AAR and IS are mostly performed manually, which can induce inter-observer variability. Exact quantification of IS is achieved by manual and subsequent digital labeling through extracting areas of the AAR and infarcted regions. Due to these conditions, the process of IS quantification can be time-intensive and prone to inter-observer variability [3], which can reduce the robustness of such studies.

Computational image analysis, assisted by training of machine learning models, has emerged as a powerful tool to enhance the precision and efficiency of cardiovascular data analysis in clinical research, notably for the segmentation of myocardial infarction using cMRI [5, 60, 65, 68]. Training and application of such machine learning models have also been used in preclinical settings to reduce inter-observer variability and the time required for analysis, in the context of stroke [4] and in particular for quantification of stroke IS by TTC staining [48]. Leveraging the ability of machine learning models and in particular of deep learning models (e.g., convolutional neural networks or U-Nets) to consistently segment complex tissue states with pixel-wise accuracy [46], might also be a promising approach to reduce inter-observer variability and increase the robustness [51] in the analysis of IS in cardioprotection research.

We here aimed to test if a surface area-related IS quantification using a deep learning segmentation model can be used for quantification of myocardial IS in pigs. For that, we retrospectively acquired image data and IS data of postmortem TTC-stained heart slices from ischemia/reperfusion (I/R) experiments in pigs for training which had been performed within the framework of studies on cardioprotection [1, 10, 11, 25–28, 35, 36, 52, 54–56]. We then developed, trained and cross-validated the trained deep learning segmentation model for identifying infarcted tissue, non-infarcted AAR tissue, non-affected (remote) tissue, right ventricular tissue and remaining areas within image data, for automation of IS quantification. The trained model was then tested on a preliminary test data set from experiments in isolated, saline-perfused rat hearts with regional I/R without/with cardioprotection.

## Methods

All procedures involving animals were conducted in accordance with the German laws for animal welfare and adhered to guidelines on the protection of animals used for scientific purposes currently in force (*American Heart Association on Research Animal Use*, adopted on November 11, 1984; *Guide for the Care and Use of Laboratory Animals*, National Institutes of Health Publication No.85-23, Revised 1996; the *guidelines from Directive 2010/63/EU of the European Parliament on the protection of animals used for scientific purposes*; ARRIVE guidelines 2.0 [43, 44]).

### Experiments in pigs

We retrospectively used published data of 390 experiments, which were performed in Göttingen and Ossabaw minipigs between 2012 and 2022. All experiments were approved by permissions from the local authorities (District of Düsseldorf G1240/11, G1407/14, G1413/14, G1610/17, G1625/17, G1655/18, G1777/20, G1868/21). IS was quantified after I/R without (*n* = 228) and with cardioprotection (*n* = 162), achieved by either mechanical interventions, i.e., local ischemic pre- [11, 27, 28] and postconditioning [1, 17, 55, 56], remote ischemic conditioning [10, 35, 36, 52] or pharmacological interventions, i.e., danegaptide and diazoxide [26, 56]. We here intentionally included such a broad variety of experiments to cover a wide range of IS from 0 to 73% of the AAR (supplemental Fig. 1).

#### Ischemia/reperfusion

**Fig. 1:**
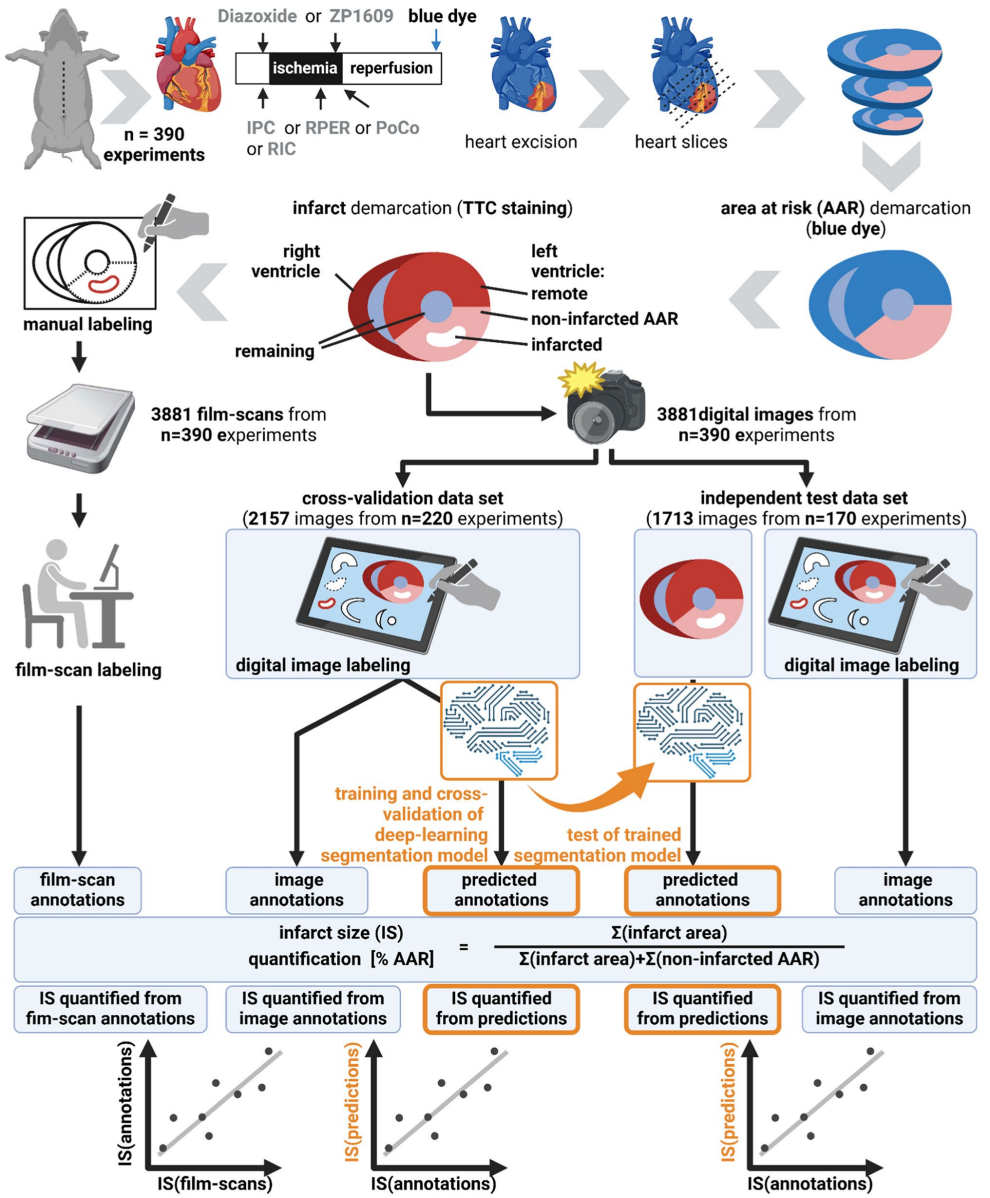
Experimental setup and pipeline for training and evaluation of a deep learning segmentation model. Top: experimental protocols, demarcation of AAR and infarcted tissue and manual labeling of transparent films. Middle: digitalization process (scans and digital images) and creating of labeled data sets for training, cross-validation and testing of a deep learning segmentation model. Bottom: quantification of IS for film-scans, image annotations and predictions and the comparison between those. *AAR* area at risk, *IPC* ischemic preconditioning, *IS* infarct size, *I/R* ischemia/reperfusion, *PoCo* ischemic postconditioning, *RIC* remote ischemic conditioning, *RPER* remote ischemic perconditioning, *TTC* triphenyl tetrazolium chloride, *ZP1609* Danegaptide. Created with BioRender.com

**Fig. 2:**
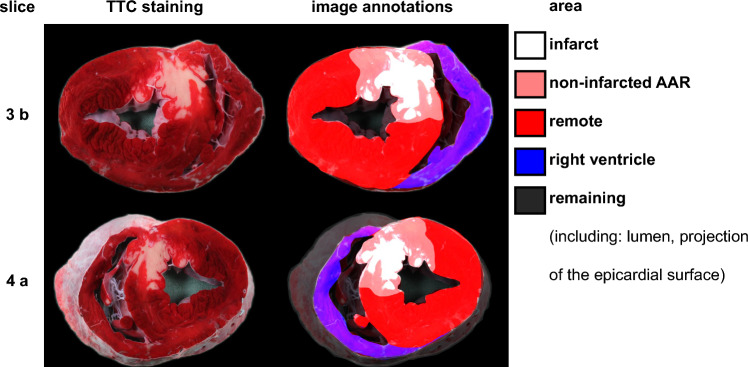
Example of segmentation areas, showing two slices (the third slice in basal orientation and the fourth slice in apical orientation) from a representative experiment. *AAR* area at risk, *TTC* triphenyl tetrazolium chloride

The basic experimental procedure of ischemia (I) and reperfusion (R) was the same in all studies. In brief, open-chest pigs were subjected to 60 min occlusion of the left anterior descending coronary artery and subsequent 3 h R without or with the above-mentioned cardioprotective interventions. The AAR did not differ between the studies (from 9 to 38% of the left ventricle, supplemental Fig. 1). IS was quantified by standard methods [3]. In brief, after R the left anterior descending coronary artery was re-occluded at the same location as during I, and a blue dye (Patentblau V, Guerbet GmbH, Sulzbach, Germany) was injected to delineate the AAR as remaining unstained, while non-affected (remote) tissue was stained blue. The heart was quickly removed from the chest and cut manually into five to seven slices of approximately 10–20 mm thickness perpendicular to the left ventricular long axis. Slices were then subjected to a second staining with TTC to demarcate infarcted from non-infarcted tissue. In the non-infarcted tissue, due to the presence of dehydrogenase enzymes, TTC is reduced to a red formazan compound, while in infarcted tissue dehydrogenase activity is lacking [9]. Thus, brick red tissue identified remote tissue, light red non-infarcted AAR and white tissue infarcted tissue (Figs. 1, 2). The respective demarcated tissue areas (apical and basal side of each slice, respectively) were manually labeled on a transparent film (Fig. 1), and the slices were weighed. Slices were additionally documented using high-resolution digital photography (again, apical and basal side of each slice, respectively). Digital images were taken under illumination, in constant distance from slice to lens, had a resolution of 5 or 18 mega pixel (DSC-H50, Sony, Tokyo, Japan, or EOS 600D, Canon, Tokyo, Japan), and were in true color (24-bit). All relevant image characteristics and stained tissue areas were clearly visible (Fig. 2, TTC-staining). However, coloration intensity and illumination conditions could vary between experiments.

#### Quantification of infarct size from film-scan annotations

Transparent films were scanned and digital labeling was used to determine the areas of the following tissues from the apical and basal slice surfaces: infarcted, AAR, remote and right ventricle (film-scan annotations, Fig. 1 left). The quantification of IS was based on the infarcted and AAR surface area in the left ventricle and normalized to the mass of each slice to counteract varying slice thicknesses. Thus, for each slice from a given experiment, the basal and apical film-scan annotations were averaged and normalized to the mass of the slice. The left ventricular masses of infarcted and non-infarcted AAR were summed up, and IS was calculated as the ratio of infarcted tissue to AAR (IS quantified from film-scan annotations, please see Fig. 1, the supplemental methods and Eq. 1 for details).

### Experiments in isolated, saline-perfused rat hearts

We used preliminary data from 27 experiments in isolated, saline-perfused rat hearts. IS was quantified after I/R without (*n* = 22) and with cardioprotection by ischemic preconditioning (*n* = 5). Male Lewis rats (250–380 g, 2.5–3.5 months, central Animal Laboratory, University of Duisburg-Essen, Germany) were sedated by an intraperitoneal injection of xylazine/ketamine (100 mg/ 10 mg/ kg body weight, belapharm, Vechta, Germany/ WDT, Garbsen, Germany) before being killed by an intraperitoneal pentobarbital injection (800 mg/kg, Narkodorm®, CP-Pharma. Burgdorf, Germany). Isolation and experimental preparation of the saline-perfused heart, the methods for the measurement of hemodynamics, inclusion and exclusion criteria, as well as the quantification of IS were standard [3] and have been described in detail previously [32–36]. Hearts were subjected to a regional I/R protocol by tightening a suture (5.0. Ethicon Inc., Norderstedt, Germany) placed around the left anterior descending coronary artery proximal to its first diagonal branch using a silicone tourniquet. After 30 min of coronary occlusion, the tourniquet was released, followed by 120 min of reperfusion. Ischemic preconditioning was induced by three cycles of 5 min/ 5 min global zero-flow I/R before regional I/R [34]. As in in vivo pig hearts, AAR was demarcated with blue dye injection after re-occlusion of the coronary artery. Hearts were cut manually into five to six slices of approximately 2 mm thickness perpendicular to the left ventricular long axis. Infarcted and non-infarcted tissue demarcation and quantification of AAR and IS corresponded to that described above for pig hearts.

### Statistics

The Kolmogorov–Smirnov test was used to test normality for all numerical data. The data are presented as means ± standard deviations (SD), or as medians with interquartile ranges (IQR). IS data of different quantification methods were presented in scatter plots with linear regression analyses and correlation coefficients (Pearson’s correlation coefficient) were calculated (OriginPro, Version 2023b. OriginLab Corporation, Northampton, USA). In addition, Bland–Altman plots were created. The divergence of the linear regression line from a line of identity was evaluated through analysis of covariance (ANCOVA, SPSS Statistics 29.0.0.0, IBM, Armonk, USA). Differences were considered significant at the level of *p* < 0.05.

## Results

### Deep learning-assisted analysis of infarct size

To develop and test the potential of a deep learning segmentation model for automation of IS quantification, the following pipeline was conceived.

### Data acquisition and image preprocessing

Only digital images of TTC-stained slices were used. A total of 3869 digital images of TTC-stained heart slices from pigs (390 hearts in five to seven slices, resulting in 2285 slices, only including cut surfaces) were included in the present study. Digital images of TTC-stained slices were pre-processed and labeled to obtain a fully digitalized data set as input for the training of the deep learning segmentation model. Potentially interfering background was removed (rembg, https://github.com/danielgatis/rembg, Daniel Gatis, 2020), and digital images were cropped by aspect ratio correction with padding. An experienced observer, who had also outlined the heart slices on transparent films, labeled also the digital images. The observer was blinded to the respective protocol by assigning a random number to each image. Labeling was performed using the “Annotate” platform of the online tool Labelbox (Labelbox Inc., San Franscisco, USA). On a tablet, image annotations were added by freehand drawing on the high-resolution digital images of TTC-stained slices. The following areas on the slice surface were labeled: infarcted area, non-infarcted AAR, remote area and right ventricular area, while background and other areas, e.g. projection of the epicardial surface or the lumina, were considered as remaining areas (Fig. 2). IS was quantified from image annotations following the same procedure as with the film-scan annotations (IS quantified from image annotations, please see Fig. 1, the supplemental methods and Eq. 1 for details).

### Training of a deep learning neural network

The used model architecture was a dynamic U-Net [39], based on fastai (version 2.7.14, https://github.com/fastai/fastai, Jeremy Howard, 2018) with PyTorch (version 2.1.2) [42] backend. We have chosen a dynamic U-Net as model architecture, because it provides an appropriate balance between detail capture and computational efficiency [39], especially for limited data sets [46]. Classical machine learning approaches (like random forests, or ensemble methods like boosted trees) have been used previously in the context of IS quantification via TTC [48]. However, we have explicitly not used these approaches, as they usually do not perform well on unseen data or data with varying image characteristics [8]. More complex model architectures such as attention mechanisms from vision transformers were also not used, as they require large data volumes to reduce the chance of overfitting [66]. A pre-trained ResNet34 model was used for the capture of image characteristics. All training and analysis steps were performed on a workstation equipped with an RTX A4500 graphical processing unit (Nvidia, Santa Clara, USA). A composite loss function was implemented, consisting of weighted cross-entropy, mean absolute error and Dice similarity coefficient (DSC) [2, 6]. For details, please see supplemental methods. Input data for the model were the high-resolution digital images and the image annotations (Fig. 2). Data were randomly split into two data sets for training and validation purposes (cross-validation data, 220 experiments) and for testing purposes (test set data, 170 experiments). Further, fivefold cross-validation was set by dividing the cross-validation data into fivefold of alternative data sets (80% for training, 20% for validation purposes). Input data were resized to a uniform size (384 × 384 pixels) to reduce input size. To estimate image quality loss after the resizing transformation, the following “perceived quality” measurements were determined: structural similarity index measure (ranging from −1 to 1, where −1 represents perfect negative correlation, 0 represents no similarity and 1 represents perfect similarity) [20, 64] and peak signal-to-noise ratio (measured in dB, values between 30 and 50 dB indicate low-quality loss for images with a bit depth of 24-bit) [20, 63]. A median structural similarity index measure of 0.985 (0.01 IQR) and a median peak signal-to-noise ratio of 39.0 (4.6 IQR) dB confirmed low-quality loss. Data were augmented by rotating, zooming, warping, altering brightness, contrast or saturation, random erasing and addition of Gaussian blur to reduce the chance of overfitting and improve the capture of distinct image characteristics [40]. Training was performed using an adaptive moment estimation optimizer with a learning rate of 0.0001, for at least 300 epochs with a batch size of 26, a dropout of 50% [58] and a weight decay of 0.5%. The best model was chosen, based on the performance in the validation set. The outputs of the deep learning segmentation model were probability maps for each segmentation area, which ranged for each pixel from 0 to 1, where a value close to 1 indicates a high confidence for a given segmentation area and a value close to 0 a low confidence. Only segmentation area with the highest probabilities were used for the following analyses (predictions). Following the training and validation of a first model on the first data set fold, training was continued in the same manner for additional models in each consecutive fold for cross-validation. To confirm the model on training-independent data, its segmentation capability was challenged with the test set data and a preliminary test data set from isolated, saline-perfused rat hearts with regional I/R.

### Infarct size quantification from predictions

Predicted annotations from the cross-validation and from the test set were used to obtain averaged and normalized masses of infarcted and non-infarcted AAR tissues as described for the image annotations. IS was quantified with the same procedure as with IS quantified from image annotations (IS quantified from predictions, please see Fig. 1, the supplemental methods and Eq. 1 for details).

### Computational efficiency

Using the hardware described above, training of the deep learning segmentation model for the cross-validation data took approximately 30 h to train. Afterward, using the trained model, a single image would take about 2 s and a typical experiment with five slices (10 images) could be processed and analyzed within 20 s. Including the time required for taking the images, the total analysis took about 90 s. In contrast, the time required for transferring the slice dimensions from one experiment to the transparent films, for scanning and for IS quantification was approximately 90 min.

### Performance evaluation metrics

For accurate IS quantification, a correct surface area determination of infarcted tissue, non-infarcted AAR tissue, remote tissue and right ventricular tissue is a prerequisite. The metrics DSC, accuracy (ACC) and average precision (AP) were used to estimate the performance of the model for IS quantification (Tables 1 and 2) [38]. Each metric is represented by a numerical value, ranging from 0 (no similarity) to 1 (perfect similarity). Metrics were computed for each individual segmented area and for all segmented areas (overall segmentation). The DSC was applied to measure the similarity and overlap between the image annotations and the predictions and reflects how well the model captures the shapes and sizes of each area (supplemental Fig. 2). ACC was used as a measure to describe the proportion of correctly predicted pixels to the total number of pixels per area (supplemental Fig. 2). For the overall segmentation, ACC computation was weighted by the amount of pixels in each area (weighted ACC). AP was used to measure the model’s performance in terms of detection and localization of each area, independently from the total area sizes (expressed as the area under the precision–recall curve). For the overall segmentation, the mean AP (mAP) was computed. To further characterize the model’s performance apart from and in addition to its suitability for IS quantification, the boundary F1 score (bF1) was determined, which measures shape delineation, to estimate the model’s ability to delineate the exact border zones of each area. For equations, please see the supplemental methods and supplemental Fig. 2.

**Table 1:** Deep learning segmentation model performance metrics for predictions from fivefold cross-validation

Segmentation area	DSC	ACC	bF1	AP
Infarcted area	0.876 ± 0.007	0.992 ± 0.001	0.211 ± 0.004	0.930 ± 0.008
Non-infarcted AAR	0.838 ± 0.008	0.975 ± 0.002	0.176 ± 0.005	0.879 ± 0.008
Remote area	0.935 ± 0.004	0.971 ± 0.002	0.199 ± 0.004	0.968 ± 0.004
Right ventricular area	0.890 ± 0.004	0.986 ± 0.001	0.219 ± 0.003	0.925 ± 0.003
Remaining area	0.980 ± 0.001	0.975 ± 0.001	0.543 ± 0.002	0.994 ± 0.001

Overall segmentation areas	Overall DSC	Weighted ACC	Overall bF1	mAP
	0.904 ± 0.02	0.980 ± 0.08	0.270 ± 0.03	0.908 ± 0.06

*AAR* area at risk, *ACC* accuracy, *AP* average precision, *bF1* boundary F1 score, *DSC* Dice similarity coefficient, *mAP* mean average precision

**Table 2:** Deep learning segmentation model performance metrics for predictions from the test set

Segmentation area	DSC	ACC	bF1	AP
Infarcted area	0.874	0.990	0.255	0.897
Non-infarcted AAR	0.811	0.972	0.220	0.847
Remote area	0.935	0.972	0.266	0.966
Right ventricular area	0.894	0.987	0.296	0.913
Remaining area	0.981	0.977	0.595	0.994

Overall Segmentation areas	Overall DSC	Weighted ACC	Overall bF1	mAP
	0.899	0.979	0.326	0.933

*AAR* area at risk, *ACC* accuracy, *AP* average precision, *bF1* boundary F1 score, *DSC* Dice similarity coefficient, *mAP* mean average precision

### Evaluation of the deep learning segmentation model for infarct size quantification

#### Suitability of image annotations for IS quantification

**Fig. 3:**
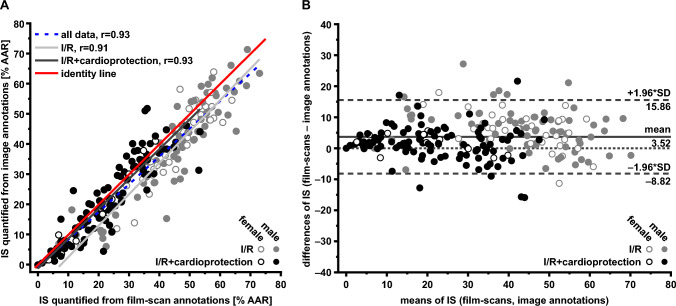
Data from infarct sizes quantified from film-scan annotations and from image annotations. **A** Linear regression analysis. **B** Bland–Altman plot. Differences and means from IS quantified from film-scan annotations and IS quantified from image annotations are plotted against one another. The solid line represents the mean of both data sets; the dashed lines represent the upper (+ 1.96-fold SD) and lower limits (− 1.96-fold SD) of agreement. *AAR* area at risk, *IS* infarct size, *I/R* ischemia/reperfusion, *r* Pearson’s correlation coefficient

To confirm the suitability and validity of the image annotations as input for a deep learning segmentation model, IS quantified from image annotations were compared with IS quantified from existing film-scan annotations (Fig. 3). An agreement between both was found (all data: *r* = 0.91, I/R data: *r* = 0.91, I/R with cardioprotection: *r* = 0.93; Fig. 3A). ANCOVA indicated no significant deviation from the line of identity. On average, the IS quantified from image annotations were estimated by 3.5% of the AAR lower than IS quantified from film-scan annotations (Fig. 3B).

#### Cross-validation

**Fig. 4:**
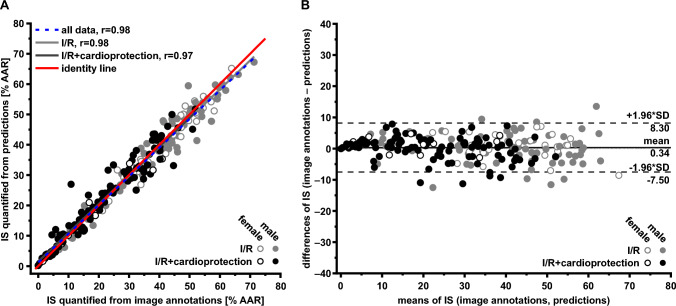
Cross-validation data from infarct sizes quantified from image annotations and from predictions. **A** Linear regression analysis. **B** Bland–Altman plot. Differences and means from IS quantified from image annotations and IS quantified from predictions are plotted against one another. The solid line represents the mean of both data sets; the dashed lines represent the upper (+ 1.96-fold SD) and lower limits (− 1.96-fold SD) of agreement. *AAR* area at risk, *IS* infarct size, *I/R* ischemia/reperfusion, *r* Pearson’s correlation coefficient

IS quantified from image annotations agreed with IS quantified from predictions (all data: *r* = 0.98, I/R data: *r* = 0.98, I/R with cardioprotection data: *r* = 0.97; Fig. 4A). ANCOVA indicated no significant deviation from the line of identity. On average, the IS quantified from predictions were estimated by 0.3% of the AAR lower than IS quantified from image annotations (Fig. 4B).

#### Test set

**Fig. 5:**
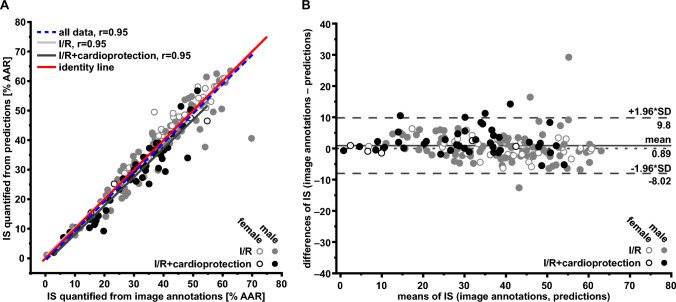
Test set data from infarct sizes quantified from image annotations and from predictions. **A** Linear regression analysis. **B** Bland–Altman plot. Differences and means from IS quantified from image annotations and IS quantified from predictions are plotted against one another. The solid line represents the mean of both data sets; the dashed lines represent the upper (+ 1.96-fold SD) and lower limits (− 1.96-fold SD) of agreement. *AAR* area at risk, *IS* infarct size, *I/R* ischemia/reperfusion, *r* Pearson’s correlation coefficient

IS quantified from image annotations agreed with IS quantified from predictions (all data: *r* = 0.95, I/R data: *r* = 0.95, I/R with cardioprotection data: *r* = 0.95; Fig. 5A). ANCOVA indicated no significant deviation from the line of identity. On average, the IS quantified from predictions were estimated by 0.9% of the AAR lower than IS quantified from image annotations (Fig. 5B). Representative examples, with high agreement between experiments (on the mean line in the Bland–Altman plot) and examples with low agreement between experiments (far from the mean line in the Bland–Altman plot) are displayed in supplemental Fig. 3A, B.

#### Test data set from isolated, saline-perfused rat hearts

**Fig. 6:**
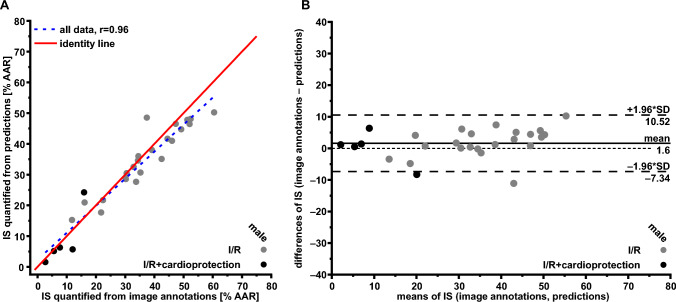
Test data set from of infarct sizes in isolated, saline-perfused rat hearts with regional ischemia/reperfusion, quantified from image annotations and from predictions. **A** Linear regression analysis. **B** Bland–Altman plot. Differences and means from IS quantified from image annotations and IS quantified from predictions are plotted against one another. The solid line represents the mean of both data sets; the dashed lines represent the upper (+ 1.96-fold SD) and lower limits (− 1.96-fold SD) of agreement. *AAR* area at risk, *IS* infarct size, *r* Pearson’s correlation coefficient

To test the deep learning segmentation model’s ability to generalize on independent unseen data, preliminary data from 27 experiments in isolated, saline-perfused rat hearts with regional I/R and subsequent TTC staining were used (282 digital images of TTC-stained heart slices, 153 slices only including cut surfaces). Rat heart size, ventricular geometry and coronary anatomy and thus the spatial extent of infarction differ from those in pigs and humans [57, 62]. Also, the ex vivo approach on isolated, saline-perfused rat hearts differs from the in vivo I/R in pigs, and the validity of TTC staining may vary between both approaches [45]. Again, an experienced observer labeled the digital images, as described above for pig hearts. IS quantified from image annotations agreed with IS quantified from predictions (all data: *r* = 0.96 Fig. 6A, B); for a representative example please see supplemental Fig. 4.

## Discussion

We have established and validated a deep learning segmentation model for IS quantification in pigs with I/R. Pigs resemble humans in cardiac and coronary anatomy, hemodynamics and the temporal and spatial progression of myocardial infarction [3, 19, 50].

There is a strong agreement between IS quantified via the deep learning segmentation model for IS quantification and the image annotations from predictions. Quantification of IS was based on the mass of infarcted and non-infarcted AAR tissue which are derived from the surface areas of the respective tissue regions. Therefore, well-segmented areas with high similarity, precision and ACC are critical. However, the model’s ability to correctly delineate the shapes in the image annotations was poor, evidenced by a low bF1. Nevertheless, a precise shape delineation is obviously not needed for accurate IS quantification.

The deep learning segmentation model saved time for IS quantification. While both manual and deep learning segmentation model approaches require demarcation of the AAR and TTC staining, initially, demarcation of regions on a transparent film and further time-intensive manual and subsequent digital labeling are not required for the deep learning-assisted IS quantification, the digital images of TTC-stained slices are sufficient. Time required for quantification is thus reduced from approximately 90 min to approximately 90 s, depending on available hardware. Such greater time efficiency is particularly of advantage for laboratories performing many experiments.

Deep learning segmentation models for IS quantification appear also useful for preclinical multi-center studies [30, 31, 47, 49], as they not only offer greater time efficiency, but also more standardized data quality and reduced inter-observer variability than manual quantification. However, image quality may vary between the participating centers, and the model may need to be validated for each participating center before use. Also, of note, the initial training of the model was based on subjectively annotated data sets, and therefore while inter-observer variability is reduced, its output is not entirely devoid of subjective variability.

The use of deep learning models improves medical diagnostics also in histopathology [61], and next-generation sequencing [67]. Deep learning for segmentation of myocardial infarction in patients, as assessed by cMRI, has already been demonstrated [5, 65]. It would be interesting to subject data from clinical trials on cardioprotective interventions in which cMRI was used to assess IS by a retrospective deep-learning based study, as done in the present study for pigs.

Using TTC to demarcate infarcted regions is also a common technique to quantify IS in small animal models, such as rats or mice, not only for myocardial infarction [36], but also for cerebral infarction [48]. To further validate the trained model, IS quantification was tested using a small preliminary data set of isolated, saline-perfused rat hearts with regional I/R. Again, there was a correlation between image annotations and predictions, suggesting a broader potential use of the developed deep learning segmentation model for IS quantification. However, in comparison to the test set data from pig experiments, the segmentation performance was inferior in terms of DSC and mAP, while the weighted ACC was still high (Table 3). Thus, the here developed deep learning segmentation model can be used for IS quantification in hearts of different size, ventricular geometry and coronary anatomy, or with different experimental setups. However, an adaptation of the model’s parameters and fine-tuning with additional image data in high quality is a prerequisite to fully transfer the described model to other species/setups.

**Table 3:** Deep learning segmentation model performance metrics for predictions from the test data set obtained in isolated, saline-perfused rat hearts

Segmentation area	DSC	ACC	bF1	AP
Infarcted area	0.743	0.961	0.131	0.743
Non-infarcted AAR	0.698	0.907	0.111	0.722
Remote area	0.704	0.850	0.058	0.700
Right ventricular area	0.288	0.958	0.035	0.145
Remaining area	0.864	0.867	0.495	0.915

Overall Segmentation areas	Overall DSC	Weighted ACC	Overall bF1	mAP
	0.659	0.909	0.166	0.654

*AAR* area at risk, *ACC* accuracy, *AP* average precision, *bF1* boundary F1 score, *DSC* Dice similarity coefficient, *mAP* mean average precision

### Limitations and future perspectives

The model focuses exclusively on quantification of IS and does not account for coronary microvascular injury, which is, besides IS, a determinant of patients’ clinical outcome [24, 41]. Training of a deep learning segmentation model with a labeled data set of high-resolution digital images of no-reflow demarcating, thioflavin-S-stained heart slices [3, 25], stand-alone or in combination with data from TTC-stained heart slices to develop an ensemble model, could be developed in the future.

The comparison between the standard method using film-scan annotations to image annotations had a deviation of 3.5% of the AAR in the Bland–Altman plot (Fig. 3B), indicating that compression or warping artifacts during outlining of the slice dimensions on the transparent films may occur with IS quantification from film scans. Since IS quantification using the raw digital image data of TTC-stained heart sections is possible, it must be used for all further examinations since such compression or warping artifacts can be excluded there.

We here used a dynamic U-Net architecture, more sophisticated models like attention-gated mechanisms used in vision transformer structures would possibly further improve the model.

## Supplementary information

Below is the link to the electronic supplementary material.

Supplementary file1 (PDF 4916 KB)

## Data Availability

Supplemental Figs. 2–4 and supplemental methods are attached as a separate pdf file.

## Code availability

Source code is available under GPL-3.0 license at https://github.com/WaywardFlex/ISQuantificationFromTTC.
