# Supplementary material for: Deep learning segmentation model for quantification of infarct size in pigs with myocardial ischemia/reperfusion
Source: Basic Res Cardiol. 2024 Sep 30;119(6):923–36. doi: 10.1007/s00395-024-01081-x (PMC11628591; doi:10.1007/s00395-024-01081-x)
Supplement: Supplementary file 1 — Supplementary file1 (PDF 4916 KB) [file 395_2024_1081_MOESM1_ESM.pdf]

Supplement

Deep learning segmentation model for quantification of infarct size in pigs with myocardial ischemia/reperfusion

Felix Braczko[1], Andreas Skyschally[1], Helmut Lieder[1], Jakob Nikolas Kather[2, 3], Petra Kleinbongard[1], Gerd Heusch[1]

[1]Institute for Pathophysiology, West German Heart and Vascular Center, University of Duisburg-Essen, Essen, Germany

[2]Department of Medicine III, University Hospital RWTH Aachen, Aachen, Germany

[3]Else Kroener Fresenius Center for Digital Health, Medical Faculty Carl Gustav Carus, Technical University Dresden, Dresden, Germany.

Supplemental methods

Quantification of infarct size

$$IS\ [\%\ AAR] = \frac{\sum_{i=1}^{n} m_{INF,i}}{\sum_{i=1}^{n} m_{AAR,i}} \times 100\%$$

with: $$m_{INF,i}[g] = m_{LV,i}[g] \times \frac{\bar{A}_{INF,i}}{\bar{A}_{INF,i}+\bar{A}_{AAR,i}}$$

with: $$m_{AAR,i}[g] = m_{LV,i}[g] \times \frac{\bar{A}_{AAR,i}}{\bar{A}_{INF,i}+\bar{A}_{AAR,i}}$$

and with: $$m_{LV,i}[g] = m_i[g] \times \frac{\bar{A}_{INF,i}+\bar{A}_{AAR,i}+\bar{A}_{remote,i}}{\bar{A}_{INF,i}+\bar{A}_{AAR,i}+\bar{A}_{remote,i}+\bar{A}_{RV,i}}$$

(Eq. 1)

Where $IS\ [\%\ AAR]$ is the infarct size per % area at risk (AAR) for an ischemia/reperfusion (I/R)±cardioprotection experiment, i is the slice of a pig heart, m is the mass in gram (g) and $\bar{A}_i$ is the average surface area of the respective tissue in slice i in mm^2 (IS quantification from scan annotations) or in pixels (image annotations or predictions). INF: infarcted tissue, LV: left ventricle, remote: viable tissue in the LV, RV: right ventricular tissue.

Neural network

A composite loss function was implemented, consisting of weighted cross-entropy (wCE), mean absolute error (L1 loss) and Dice similarity coefficient (DSC).

$$wCE = -\sum_{i=1}^{C} w_i\ [y_i\ \log(\widehat{y_i}) + (1 - y_i)\ \log(1 - \widehat{y_i})]$$

(Eq. 2)

Where C is the number of classes (here segmentation areas), w_i is the weight for segmentation area i, y_i is the ground truth of a pixel as a binary classificator for segmentation area i, and $\widehat{y_i}$ is the predicted probability of segmentation area i.

$$L1\ loss = \frac{1}{N}\sum_{i=1}^{N} |p_i - g_i|$$

(Eq. 3)

$$DSC = 1 - \frac{2\sum_{i=1}^{N} p_i g_i}{\sum_{i=1}^{N} p_i^2 + \sum_{i=1}^{N} g_i^2}$$

(Eq. 4)

Where N is the total number of pixels, p_i is the predicted probability for a respective pixel i, and g_i is the actual belonging of pixel i to a respective segmentation area (ground truth).

The loss function was then expressed as:

$$loss_{total} = wCE + L1\ loss + \frac{1}{2}\ DSC$$

(Eq. 5)

A dropout of 50% and a weight decay of 0.5% was incorporated to decrease the chance of overfitting [2], as well as a reduction of learning rate under plateau conditions. The output of the model were probability maps for each segmentation area ranging from 0 to 1 for each pixel, where a value close to 1 indicates a high confidence for a given class and a value close to 0 a low confidence. Five-fold cross-validation was performed by dividing the data randomly into data sets for training, validation and testing purposes (80% training, 20% validation). Further, five-fold cross-validation was performed by dividing the training and validation data set into in five alternative data sets (fold).

Performance evaluation

To evaluate the performance of the deep learning model, the proportion of correctly predicted pixels, the weighted pixel accuracy, the boundary F1 score and average precision were calculated. The Dice similarity coefficient (DSC) was calculated to describe the similarity between true pixel masks and predicted pixel masks. All metrics were calculated first per segmentation area, then over all segmentation areas.

DSC per segmentation area:

$$DSC(C) = \frac{2 \times |P_C \cap T_C|}{|P_C| + |T_C|}$$

(Eq. 6)

Where P_C is the set of pixels predicted as segmentation area C, T_C is the set of pixels actually belonging to segmentation area C, and $\cap$ denotes the intersection operator.

DSC overall:

$$DSC_{overall} = \frac{1}{N}\sum_{i=1}^{N} DSC(C_i)$$

(Eq. 7)

Where N is the total number of segmentation area.

Pixel accuracy per segmentation area:

$$ACC(C) = \frac{|P_{C=T_C}|}{|T_C|}$$

(Eq. 8)

Where $P_{C=T_C}$ is the count of pixels for which the predicted area matches the actual area for segmentation area C.

Weighted Pixel accuracy:

$$wACC = \sum_{i=1}^{N} w_i \times Acc.(C_i) \quad \text{with: } w_i = \frac{|T_{C_i}|}{total\ pixels}$$

(Eq. 9)

Where w_i is the weight of segmentation area i, representing its proportion of the total pixels.

Average precision:

$$AP(C) = \int_0^1 precision_C(recall_C)dR$$

$$\text{with: } precision_C = \frac{TP_C}{TP_C + FP_C}$$

$$\text{and with recall: } recall_C = \frac{TP_C}{TP_C + FN_C}$$

(Eq. 10)

Where TP_C are true positives, which are the correctly predicted pixels for the respective segmentation area C, FP_C are false positives, which are the pixels wrongly predicted as the respective segmentation area C, and FN_C are false negatives, which are the pixels that were

not predicted as the respective segmentation area C. $P_C(R_C)$ is the precision at recall threshold R_C for the respective segmentation area C.

Mean average precision:

$$mAP = \frac{1}{N}\sum_{i=1}^{N} AP(C_i)$$

(Eq. 11)

Boundary F1 Score:

$$bF1 = \frac{2 \times P_{C_b} \times R_{C_b}}{P_{C_b} + R_{C_b}}$$

(Eq. 12)

Where P_{C_b} is the precision of the boundaries of the respective segmentation area C, and R_{C_b} is the recall of the boundaries of the respective segmentation area C.

Overall boundary F1 Score:

$$bF1_{overall} = \frac{1}{N}\sum_{i=1}^{N} bF1(C_i)$$

(Eq. 13)

References:

1. Paszke A, Gross S, Massa F, Lerer A, Bradbury J, Chanan G, Killeen T, Lin Z, Gimelshein N, Antiga L, Desmaison A, Köpf A, Yang E, DeVito Z, Raison M, Tejani A, Chilamkurthy S, Steiner B, Fang L, Bai J, Chintala S (2019) PyTorch: An imperative style, high-performance deep learning library. doi:10.48550/arXiv.1912.01703

2. Srivastava N, Hinton G, Krizhevsky A, Sutskever I, Salakhutdinov R (2014) Dropout: A simple way to prevent neural networks from overfitting. J Mach Learn Res 15: 1929-1958

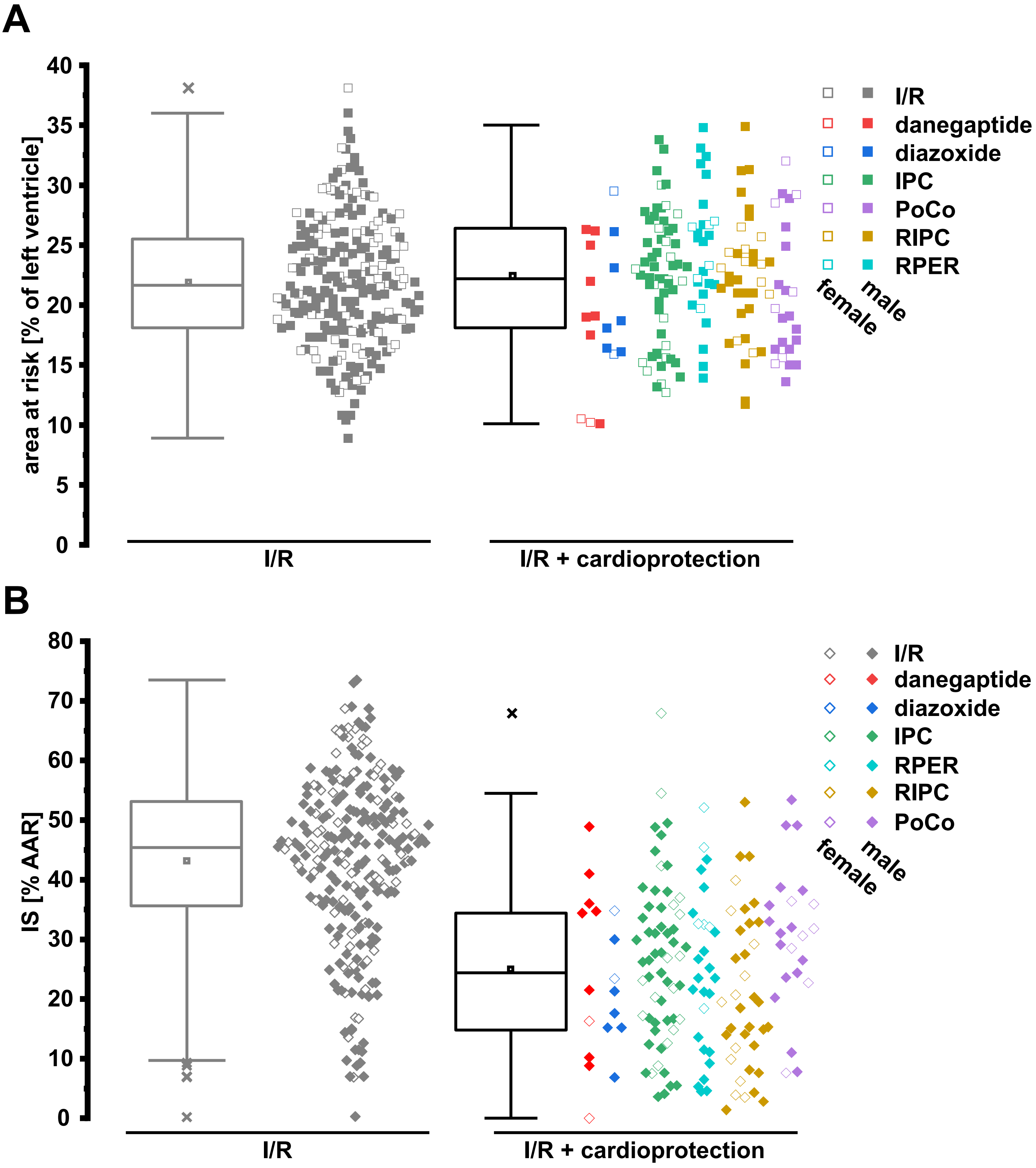

Supplemental Figure 1: Areas at risk (AAR) per left ventricle (A) and infarct sizes (IS) per AAR (B) from experiments with ischemia/reperfusion without and with additional cardioprotection.

Data shown corresponds to IS quantified from scan annotations and originates from already published studies in pigs for ischemic preconditioning (IPC), ischemic postconditioning (PoCo), remote ischemic conditioning (RIC), remote ischemic perconditioning (RPER), danegaptide and diazoxide. Data are presented as box plots with the median (line), means (square), interquartile ranges (box), 1.5*standard deviations (whisker) and outliers (cross) and as single data points.

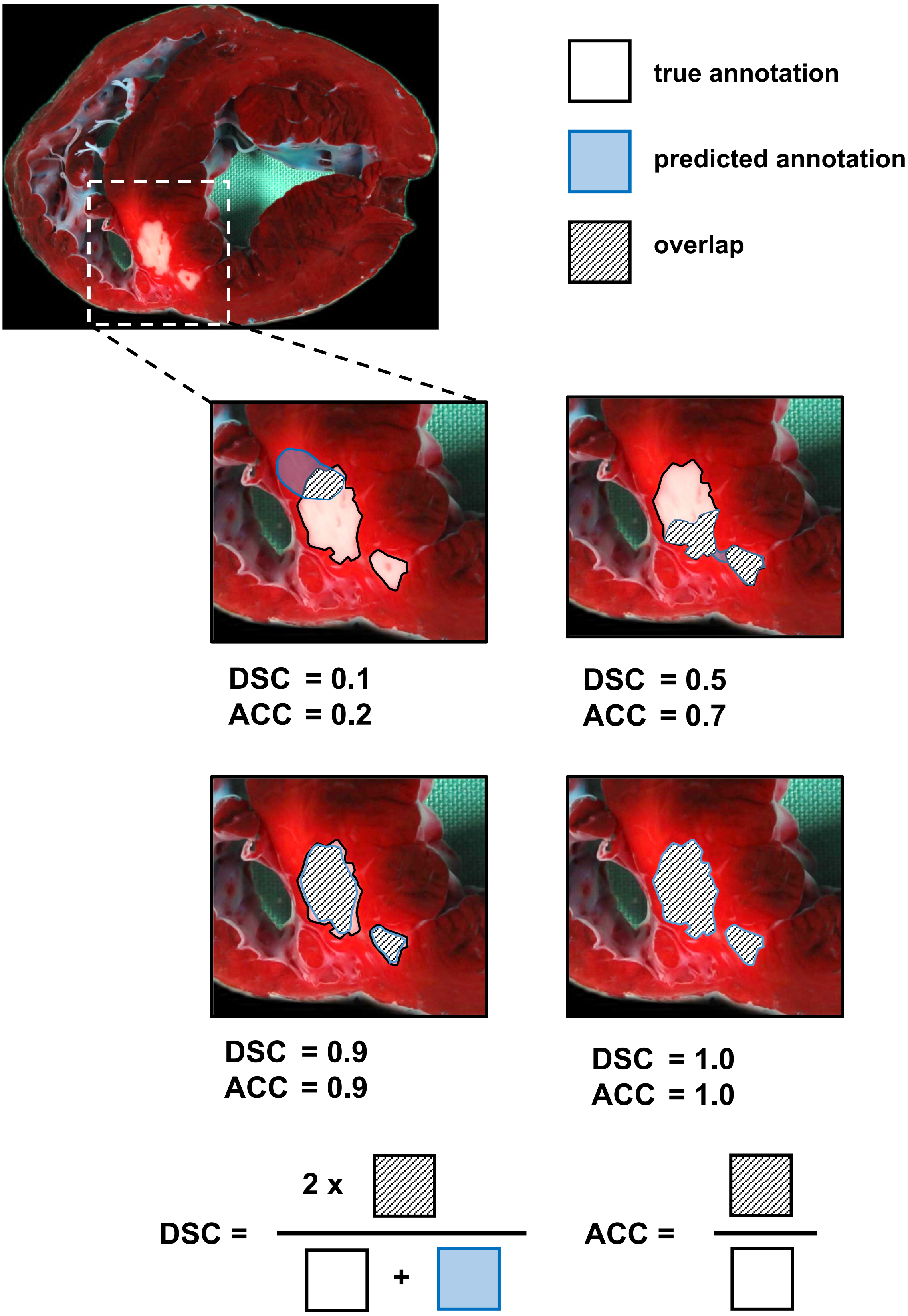

Supplemental Figure 2: Examples for low, medium and high Dice similarity coefficients (DSC) and accuracies (ACC) and their calculation. True pixel mask indicates annotations used as input for a deep learning instance segmentation model and predicted pixel-mask indicates its output. With the overlap of both, DSC is calculated as a performance metric for a deep learning model.

Supplemental Figure 3 A

IS [% AAR] quantified from film-scans: **32.1%**
IS [% AAR] quantified from image annotations: **32.5%**
IS [% AAR] quantified from predictions: **32.3%**

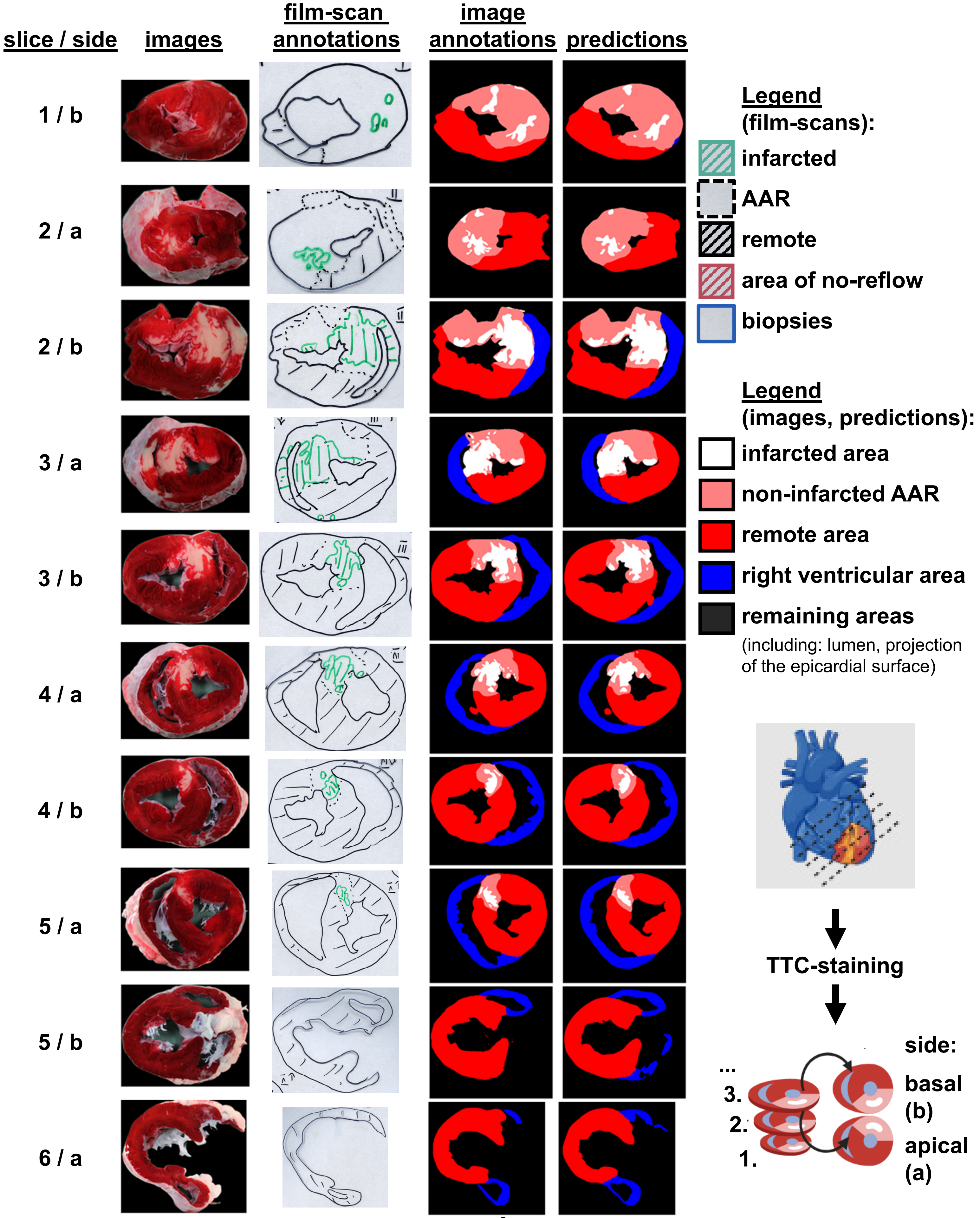

Supplemental Figure 3 B

IS [% AAR] quantified from film-scans: **42.3%**
IS [% AAR] quantified from image annotations: **44.7%**
IS [% AAR] quantified from predictions: **33.1%**

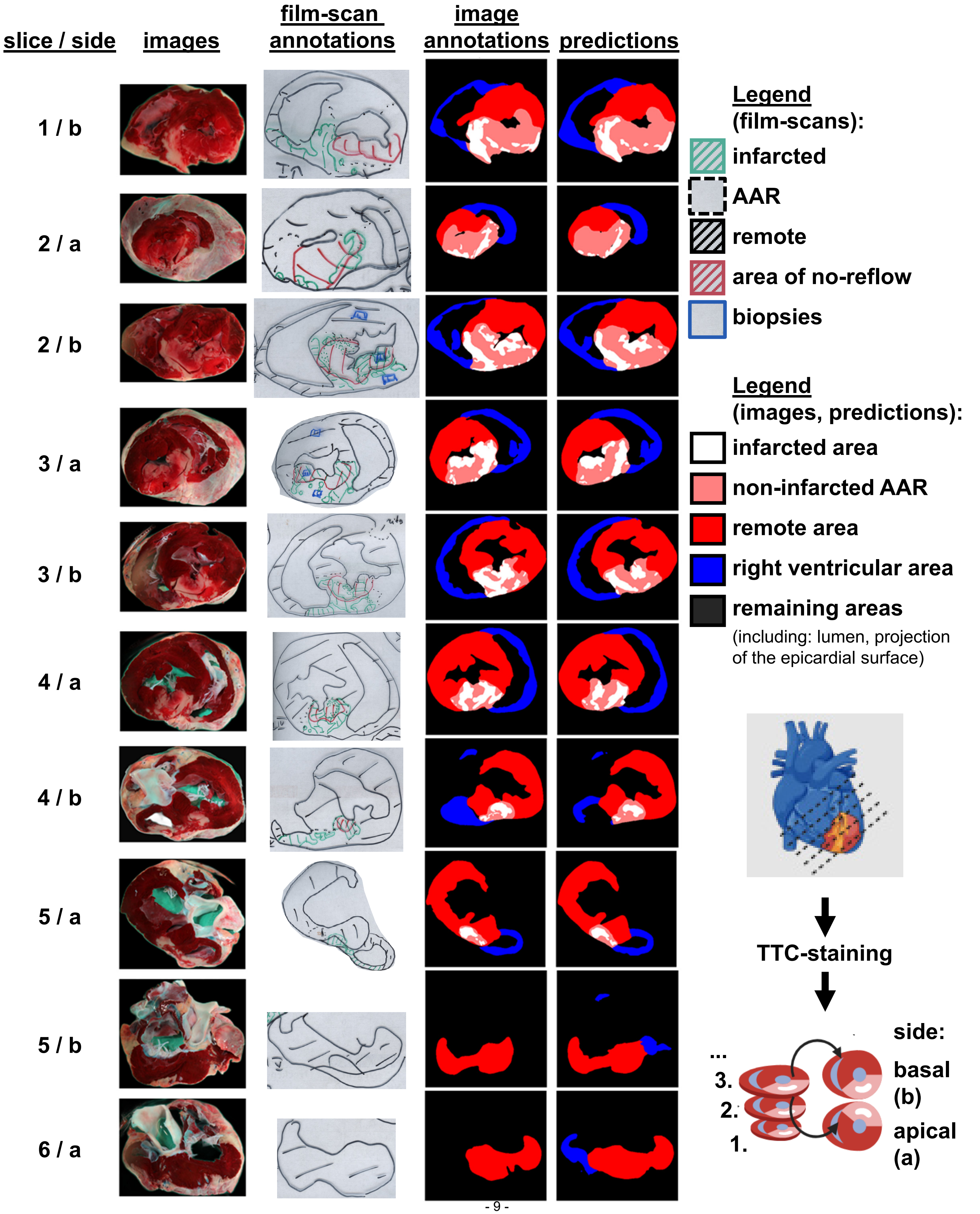

Supplemental Figure 3: Examples of digital images, film-scan annotations, image annotations and predictions as inputs and outputs from the deep learning segmentation model. Digital images of TTC-stained pig heart slices from representative experiments (digital images, film-scan annotations, image annotations and the deep learning model's predictions). A: example with high agreement between IS quantified from film-scans, image annotations and predicted annotations. B: example with low agreement between manually quantified IS and IS calculated from predictions. Additional annotations are shown on the film-scans (area of no-reflow, biopsies), which were not defined separately in the deep learning segmentation model. Film-scan annotations include infarcted area (green, crosshatched), AAR (black, dashed border), remote area (black, crosshatched), area of no-reflow (red, crosshatched) and lesions from biopsies (blue). Image annotations and predictions included infarcted area (white), non-infarcted AAR (light red), remote area (dark red), right ventricular area (blue) and remaining areas (black). AAR: area at risk, IS: infarct size, TTC: triphenyl tetrazolium chloride.

Supplemental Figure 4

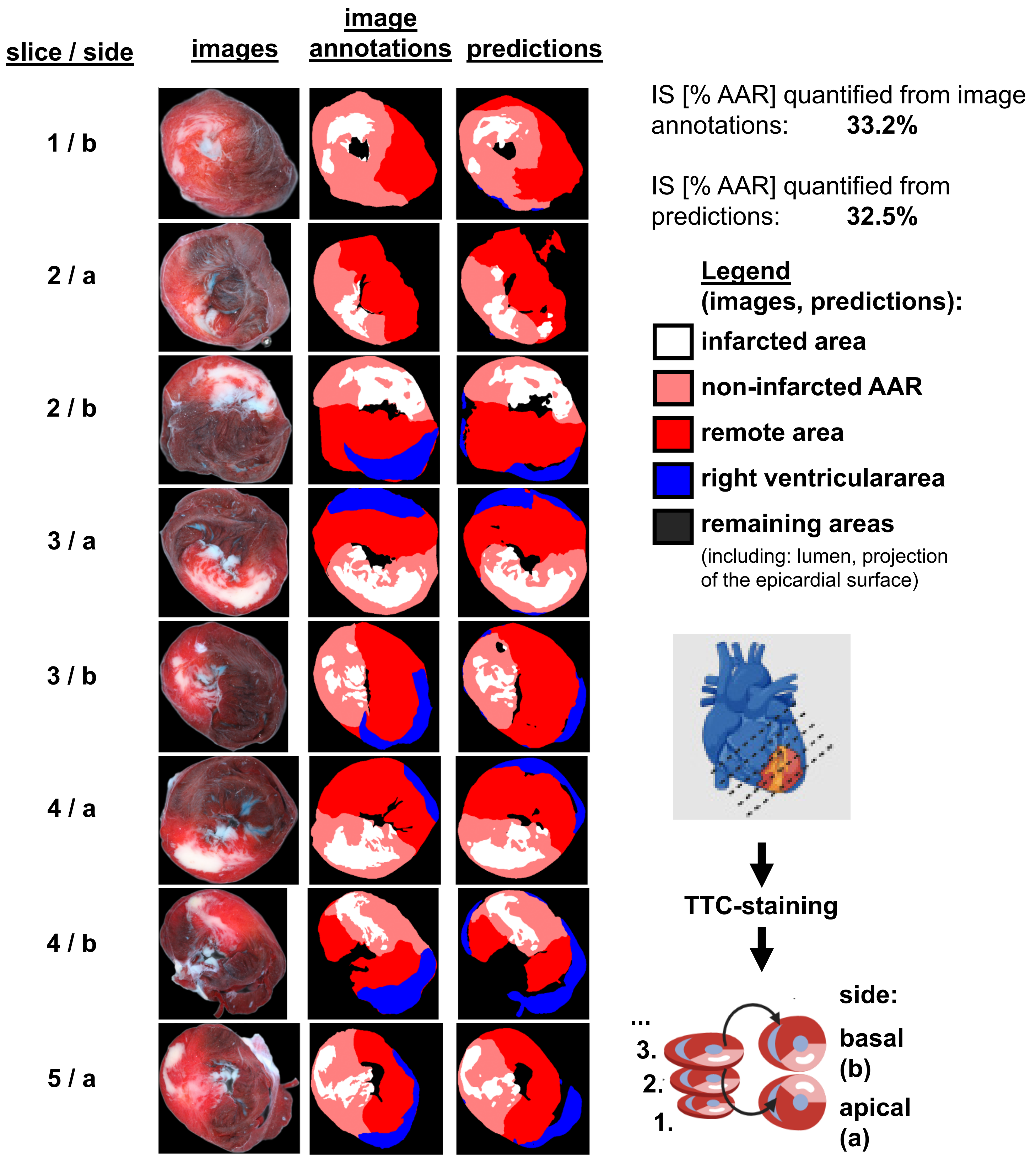

Supplemental Figure 4: Examples of digital images, image annotations and predictions from a test data set of preliminary experiments in isolated saline perfused rat hearts as a additional test data set. Digital images of slices from rat hearts with regional ischemia reperfusion from representative experiments (digital images, film-scan annotations, image annotations and the deep learning model's predictions). Image annotations and predictions included infarcted area (white), non-infarcted AAR (light red), remote area (dark red), right ventricular area (blue) and remaining areas (black). AAR: area at risk, IS: infarct size.
